# Family matters: influenza vaccination uptake and associated factors in children aged 6 months–6 years

**DOI:** 10.3389/fpubh.2025.1704497

**Published:** 2025-12-19

**Authors:** Shirley Shapiro Ben David, Shiraz Vered, Limor Adler, Daniella Rahamim Cohen, Afif Nakhleh, Yulia Binyaminov, Michal Stein, Joseph Azuri

**Affiliations:** 1 Maccabi Healthcare Services, Division of Health, Tel Aviv, Israel; 2 Gray Faculty of Medical and Health Sciences, Tel Aviv University, Tel Aviv, Israel; 3 Maccabi Healthcare Services, Haifa, Israel; 4 Institute of Endocrinology, Diabetes and Metabolism, Rambam Health Care Campus, Haifa, Israel; 5 Azrieli Faculty of Medicine, Bar-Ilan University, Safed, Israel; 6 Pediatric Infectious Disease Unit, The Edmond and Lily Safra Children's Hospital, Chaim Sheba Medical Center, Ramat Gan, Israel

**Keywords:** children, influenza, vaccination, predictors, family, uptake

## Abstract

**Background:**

Influenza poses significant health risks to young children, who are more vulnerable to severe complications. Despite effective vaccines, influenza vaccination rates among children are suboptimal. This study aims to identify the key predictors of influenza vaccine uptake among children aged 6 months to 6 years.

**Methods:**

A retrospective cohort study used data from Maccabi Healthcare Services in Israel. Electronic medical records provided demographics, vaccination status, comorbidities, healthcare use, and parental vaccination. Logistic regression identified factors associated with influenza vaccine uptake.

**Results:**

Among 293,078 children aged 6 months to 6 years during the 2022–2023 flu season, only 41,885(14.3%) received the influenza vaccine. Patients with higher socioeconomic status were more often vaccinated (51.1% vs. 26.4%, *p* < 0.001). Parental smoking was inversely associated with more non-smoking parents in the vaccinated group (72.6%) compared to the unvaccinated (66.1%, *p* < 0.001). The factor most strongly associated with child vaccination was parental vaccination status (OR 41.86, 95% CI [40.54–43.22], *p* < 0.001). Other significant predictors were higher socioeconomic status (OR = 2.29, 95% CI [2.15–2.43], *p* < 0.001) and frequent healthcare utilization, such as a high rate of doctor visits during the preceding year (four or more visits: OR = 2.09, 95%, CI [1.90–2.29], *p* < 0.001). Conversely, children from minority populations and those with parents who smoked were less likely to be vaccinated (one smoking parent, OR = 0.85, 95% CI [0.82–0.88], *p* < 0.001; and two smoking parents, OR = 0.80, 95% CI [0.74–0.87], *p* < 0.001). For children aged 18 months to 6 years (*n* = 247,381), vaccination in the previous year was also a significant predictor (OR 10.35, 95% CI [9.97–10.74]).

**Conclusion:**

The study highlights the critical role of parental vaccination status in influencing child vaccination rates. A family-centered approach to vaccination promotion can enhance vaccine coverage among young children, contributing to better health outcomes and robust community immunity.

## Introduction

Influenza is usually an acute, self-limited, and uncomplicated disease in healthy children. However, it poses significant health risks, particularly for young children, who are more vulnerable to severe complications (1, 2). Moreover, children, particularly those in elementary school and younger, are a major driver for the spread of influenza within local communities (3). In addition, Influenza is also a significant contributor to absenteeism in the workplace and schools (4, 5).

Two influenza vaccines are recommended for children: inactivated and live attenuated (6). The inactivated influenza vaccine (IIV) is administered intramuscularly, and the main contraindication is a history of severe allergic reaction to a previous flu vaccine or its components. The live attenuated influenza vaccine (LAIV) is given as a nasal spray. It is contraindicated in children with immunosuppression, asthma, or recent wheezing; those receiving aspirin therapy; those with active cerebrospinal fluid leaks; those who have recently taken specific influenza antivirals; or anyone with a history of a severe allergic reaction to a previous flu vaccine or its components.

Both IIV and LAIV are highly effective and significant tools for preventing virus spread and protecting at-risk populations (7, 8). However, their effectiveness can vary based on the influenza season, the circulating strains, and the age group of the children (9, 10). During the 2022–23 influenza season, influenza vaccination in children aged 6 months to 6 years was moderately to highly effective: vaccine effectiveness against medically attended influenza illness ranged from approximately 50 to 71%, and vaccine effectiveness against influenza-associated hospitalization was approximately 50 to 68% among vaccinated children (11–14). Evidence strongly supports that widespread immunization of children contributes to herd immunity and reduces influenza infection rates among unvaccinated children and adults (15). Many studies have found Influenza vaccines to be safe in children and adults (16, 17).

According to the Israeli Ministry of Health, all children 6 months or older should receive the influenza vaccine (6). IIV is recommended from the age of 6 months, and LAIV is recommended for children without contraindications from the age of 2 years. Children aged 6 months to 8 years receiving the influenza vaccine for the first time require two doses during their initial influenza season. Influenza vaccines are provided free of charge.

Despite the availability of safe and effective vaccines, influenza vaccination rates among children often fall short of public health targets (18, 19). Understanding the predictors of influenza vaccine uptake among children is crucial for public health efforts aimed at reducing the burden of influenza-related illnesses. Previous research has identified various factors that can influence vaccination behavior in children (20). These include parental beliefs and attitudes toward vaccines, healthcare provider recommendations, socioeconomic status, and access to healthcare services (21). Additionally, demographic factors such as age, socioeconomic status, ethnicity, and chronic health conditions have been shown to influence vaccine uptake (22). However, the relative importance of these factors can vary across different populations and settings, making it essential to study predictors of vaccination in these contexts.

The study aims to identify and analyze key predictors of influenza vaccine uptake among children aged 6 months to 6 years. The focus on this age group was chosen to better understand early-life factors influencing vaccination, as Israel’s school-based influenza vaccination program begins only in second grade. Specifically, we examined the impact of parental vaccination status, chronic health conditions, and demographic characteristics on vaccination uptake in this population. This information is vital for designing effective interventions that can increase vaccine coverage and protect children from the dangers of influenza.

## Methods

### Study design and settings

A retrospective cross-sectional study was conducted in Maccabi Healthcare Services (MHS). MHS is Israel’s second-largest health maintenance organization (HMO). It serves approximately a quarter of the Israeli population nationwide and has over 2.6 million members. The MHS member population is representative of the broader Israeli population, and all member data is stored in a centralized database.

All MHS members are encouraged to receive influenza vaccines. At the beginning of each flu season, a targeted campaign is launched to encourage the HMO’s members, including children, to get vaccinated. The campaign includes text messages to parents and emails. Vaccines are administered mainly by nurses or physicians in MHS local clinics. All vaccine records are in MHS’s unified database.

### Study population

All children aged 6 months to 6 years who were members during the influenza season of 2022–2023 (September 2022–March 2023) were included.

### Variables

Data from the electronic medical records of children aged 6 months to 6 years were collected from the extensive MHS database.

We extracted, for each child, demographic characteristics, including socioeconomic status (SES) and sector (Arab, Ultra-Orthodox Jews, or general Jews), based on patients’ addresses and categorized according to the Israeli Central Bureau of Statistics (23).

Comorbidities were identified according to the methodology of the MHS chronic registries, which employ an algorithm that integrates clinical and administrative data, including diagnostic codes, laboratory findings, imaging results, prescribed medications, and hospital discharge documents, to detect patients with chronic illnesses (24). The presence of chronic disease was identified by a positive registry record during the study period. Chronic diseases included homebound, diabetes, hypertension, and inflammatory bowel disease.

Influenza vaccination status was defined as receipt of one or more influenza vaccines during the 2022–2023 season (September 1, 2022, to March 31, 2023). For children aged 18 months or older, vaccination status from the 2021–2022 season (September 1, 2021 to March 31, 2022) was also collected.

Data on hospitalizations and medical encounters were collected from the preceding year (September 2021–August 2022).

According to the database records, each child was linked to their parents. Parental influenza vaccination status for the 2022–2023 season (September 1, 2022 to March 31, 2023) was obtained accordingly.

### Statistical analysis

Categorical data were reported as the number and percentage (%) and compared using the Chi-square test. Continuous variables were reported as mean ± standard deviation (SD), and between-group comparisons were performed using the T-test. Logistic regression analysis was used to predict influenza vaccine uptake. All covariates were entered into the model simultaneously, with no variable selection procedures applied. SES and ethnicity had very low levels of missing data (approximately 0.4 percent), which appeared to be missing at random. Parental smoking status had about 18 percent missing data, but these were unrelated to any measured variables. Complete case analyses were therefore considered unbiased, and the large sample size limited concerns about loss of statistical power. In contrast, the variable indicating vaccination in the previous year had roughly 15 percent missingness, which was not missing at random because children under 18 months did not have preceding-year data. A secondary model was estimated for children aged 18 months or older, for whom information on vaccination in the preceding year was available. This covariate was included only in that age-restricted model.

All analyses were conducted using SPSS version 29. *p* values of 0.05 or less were considered statistically significant.

The manuscript adhered to the STROBE guidelines for reporting observational studies in epidemiology (25).

### Ethical approval

Ethical approval for the study was obtained by the MHS Institutional Review Board, 0150-22-MHS. Informed consent was waived due to the anonymization of all data, which were extracted solely from electronic health records within the database.

## Results

During the 2022–2023 flu season (September to March), 41,885 (14.3%) children aged 6 months to 6 years received the influenza vaccine. Their mean (SD) age was 3.9 (2) years, and 51.4% were males. Among the vaccinated children, 35,068 received IIV, and 6,817 received LAIV.

### Univariate analysis

Vaccination status was associated with several socioeconomic, demographic, and health-related factors (Table 1). Patients with higher socioeconomic status were more likely to be vaccinated, with 51.1% of vaccinated individuals in the high socioeconomic status group compared to 26.4% of unvaccinated individuals (*p* < 0.001). Ethnicity also played a significant role, as the vaccinated group included fewer individuals from minority populations (Arabs, 2.8%; ultra-orthodox Jews, 7.2%, *p* < 0.001) compared to the unvaccinated group (Arabs, 7.8%; ultra-orthodox Jews, 19.9%; *p* < 0.001). Parental smoking status was inversely associated with vaccination, with higher rates of non-smoking parents in the vaccinated group (72.6%) compared to the unvaccinated group (66.1%; *p* < 0.001).

**Table 1:** Demographic, socioeconomic, parental, and health-related characteristics of vaccinated and unvaccinated children.

Variable	No vaccine *N* = 251,193 (85.7%)	Vaccine *N* = 41,885 (14.3%)	*p*-value^1^
Age	3.8 ± 1.9	3.9 ± 2.0	<0.001
Sex (male)	128,526 (51.2)	21,533 (51.4)	0.356
Socioeconomic status^2^, N	250,106 (99.6)	41,677 (99.6)	
1–4 (Low)	76,969 (30.8)	4,991 (12.0)	<0.001
5–7 (Med)	107,219 (42.9)	15,376 (36.9)	
8–10 (High)	65,918 (26.4)	21,310 (51.1)	
Sector, N	250,417 (99.7)	41,747 (99.7)	
Arab	19,531 (7.8)	1,183 (2.8)	<0.001
Orthodox Jewish	49,739 (19.9)	2,995 (7.2)	
Other	181,147 (72.3)	37,569 (90.0)	
Parent smoking status, N	203,637 (81.1)	36,715 (87.7)	
No	134,518 (66.1)	26,668 (72.6)	<0.001
One parent	60,649 (29.8)	8,990 (24.5)	
Two parents	8,470 (4.2)	1,057 (2.9)	
Comorbidities^3^			
Any	187 (0.1)	61 (0.1)	<0.001
Parental vaccination ^4^			
No	236,589 (94.2)	9,747 (23.3)	<0.001
One parent	13,422 (5.3)	21,020 (50.2)	
Two parents	1,182 (0.5)	11,118 (26.5)	
Health Service Usage in the preceding year			
Number of in person pediatrician/primary care visits			
0	27,049 (10.8)	2,569 (6.1)	<0.001
1–3	84,668 (33.7)	12,263 (29.3)	
4+	139,476 (55.5)	27,053 (64.6)	
Number of remote (telephone and/ or digital) pediatrician/primary care visits			
0	66,815 (26.6)	4,762 (11.4)	<0.001
1–3	92,381 (36.8)	13,549 (32.3)	
4+	91,997 (36.6)	23,574 (56.3)	
Total number of pediatrician/primary care visits			
0	16,306 (6.5)	885 (2.1)	<0.001
1–3	19,309 (19.6)	5,118 (12.2)	
4+	185,578 (73.9)	35,882 (85.7)	
Hospitalization	15,348 (6.1)	2,731 (6.5)	0.001
Vaccine information^5^ N	212,172 (84.5)	35,209 (84.1)	
Flu Vaccine in the Previous Season	14,990 (7.1)	22,600 (64.2)	<0.001

1 Chi-square test or Fisher’s exact test for categorical variables and T-test for age.

2 Socioeconomic status defined by the Israel Central Bureau of Statistics (23).

3 Comorbidities, as documented in the MHS registries, include homebound status, diabetes, hypertension, and inflammatory bowel disease; see Supplementary Table 1.

4 Parental influenza vaccination status for the 2022–2023 season (September 1, 2022, to March 31, 2023).

5 Include children aged over 18 months.

Healthcare utilization and vaccination history were strong predictors of vaccination. Patients who received the flu vaccine in the previous season (57.4% vs. 6.2%, *p* < 0.001) or whose parents were vaccinated (both parents: 26.5% vs. 0.5%, one parent: 50.2% vs. 5.3%; *p* < 0.001) were significantly more likely to be vaccinated. Additionally, frequent healthcare utilization was associated with higher vaccination rates: 64.6% of vaccinated individuals had 4 or more pediatrician or primary care physician visits, compared with 55.5% of unvaccinated individuals (*p* < 0.001).

### Factors associated with influenza vaccination among children, 2022–2023, multivariable analysis children aged 6 months to 6 years (*n* = 293,078)

**Figure 1:**
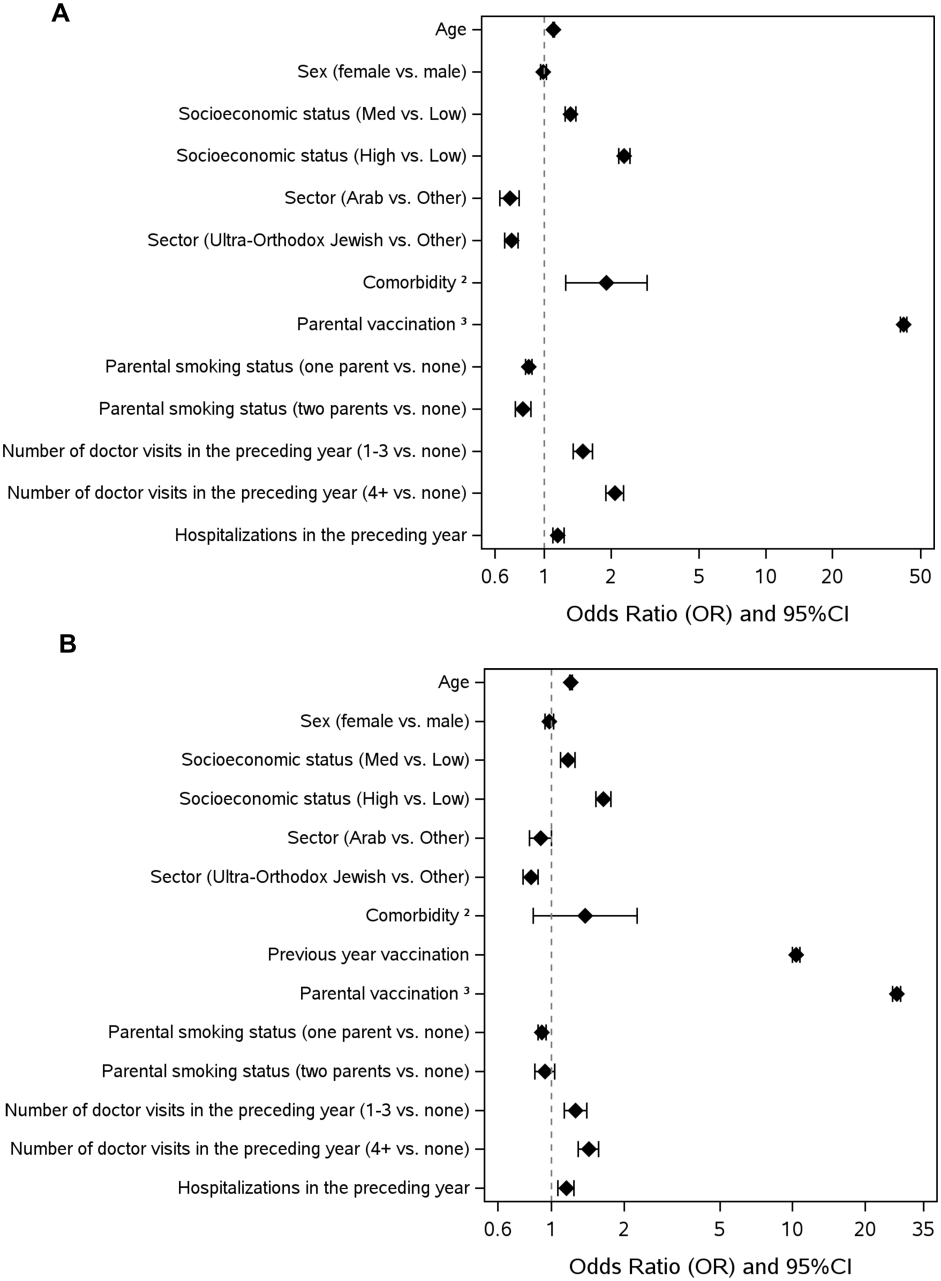
**(A)** Factors influencing influenza vaccine uptake, children aged 6 months to 6 years (*n* = 293,078). **(B)** Factors influencing influenza vaccine uptake, children aged 18 months- 6 years (*n* = 247,381). Odds ratios (OR) and 95% confidence intervals for various factors influencing vaccination uptake. The dashed line represents the reference value of OR = 1. Socioeconomic status defined by the Israel Central Bureau of Statistics (23); comorbidities, as documented in the MHS registries, include homebound status, diabetes, hypertension, and inflammatory bowel disease; parental vaccination in the same influenza season, 09/2022–03/2023; total number of pediatrician or primary care doctor visits registered in MSH in the year before the study, in person, by telephone, and/or digital encounters; hospitalizations in the preceding year.

Several factors significantly influence vaccine uptake in children aged 6 months to 6 years (Figure 1A; Supplementary Table 2). Parental vaccination was strongly associated with vaccine uptake (OR = 41.86, 95% CI [40.54–43.22], *p* < 0.001) and with high socioeconomic status (OR = 2.29, 95% CI [2.15–2.43], *p* < 0.001). Comorbidities significantly increased the likelihood of vaccination (OR = 1.99, 95% CI [1.31–3.02], *p* < 0.001), as did frequent doctor visits in the preceding year (1–3 visits: OR = 1.49, 95% CI [1.35–1.65], *p* < 0.001; 4 + visits: OR = 2.09, 95% CI [1.90–2.29], *p* < 0.001). Conversely, minorities had lower odds of vaccine uptake compared to others (Arab vs. Other: OR = 0.70, 95% CI [0.63–0.77], *p* < 0.001; Ultra- Orthodox Jewish vs. Other: OR = 0.71, 95% CI [0.66–0.76], *p* < 0.001) and parental smoking status negatively impacted vaccination, with one smoking parent (OR = 0.85, 95% CI [0.82–0.88], *p* < 0.001) and two smoking parents (OR = 0.80, 95% CI [0.74–0.87], *p* < 0.001).

### Children aged 18 months to 6 years (*n* = 247,381), including prior-season vaccination covariate

The most prominent factor associated with vaccination was parental vaccination (OR 27.06 95% CI [26.02–28.13], *p* < 0.001) (Figure 1B; Supplementary Table 3). Another substantial factor was vaccination in the previous year (OR 10.35 95% CI [9.97–10.74], *p* < 0.001). Higher socioeconomic status and a higher rate of doctor visits during the preceding year were associated with vaccination (OR 1.64, 95% CI [1.53–1.76] and OR 1.43, 95% CI [1.30–1.59], *p* < 0.001, respectively).

## Discussion

In this large retrospective cohort of 293,078 children aged 6 months to 6 years, influenza vaccine uptake was low (41,885; 14.3%). Parental vaccination clustered strongly with child vaccination: among vaccinated children 50.2% had one vaccinated parent and 26.5% had two vaccinated parents versus 5.3 and 0.5%, respectively, among unvaccinated children, and parental vaccination was the strongest predictor in multivariable models (OR 41.86, 95% CI 40.54–43.22). Higher socioeconomic status and frequent prior healthcare utilization were also positively associated with child vaccination, while parental smoking and minority status were inversely associated. Prior child-year vaccination also strongly correlated in the 18-month–6-year subgroup (aOR 10.35, 95% CI 9.97–10.74). This finding may suggest that parental behavior and attitudes toward vaccination play a critical role in determining whether young children receive vaccinations.

The overall influenza vaccination coverage among young children in our cohort (14.3% in 2022–23) is markedly low. The Israeli Ministry of Health recommends routine annual influenza vaccination for all children aged ≥6 months (6). The World Health Organization and recent global reviews recommend substantially higher coverage to ensure both direct protection of children and community-level benefits (19). In comparison, during the 2022–2023 influenza season, the coverage rate for influenza vaccination among children aged 6 months to 6 years in the United States was approximately 55% (12). This remains below the Healthy People 2030 target of 70% and lower than pre-pandemic levels. Globally, a systematic review and meta-analysis found that seasonal influenza vaccine uptake among children aged 6–59 months was 41% (95% CI: 33–50%) across multiple countries (26). A review of national influenza policies also highlights major differences in implementation and program reach across WHO member states (19). These comparisons suggest that while low pediatric influenza uptake is not unique to Israel, the coverage observed lies at the lower end of the reported spectrum and represents a pressing local public health priority.

The influence of parental vaccination status on child vaccination rates likely stems from multiple factors: parents who vaccinate themselves tend to have greater trust in vaccines and the healthcare system, lower hesitancy, and better awareness of vaccine benefits, which promotes timely vaccination for their children and models positive health behaviors (27); families may also coordinate vaccinations during the same visit or respond together to reminders and outreach. Our findings align with prior research on the central role of parental attitudes and behaviors in child vaccine uptake and with a previous MHS study showing concordant vaccination decisions within families (20, 28–30). However, the very large magnitude of the parental–child association probably reflects a combination of actual parental influence, residual confounding by unmeasured family-level factors such as health literacy, access, vaccine confidence, logistical clustering (same-visit vaccination, shared reminders or outreach), differential recording, and potential estimation artifacts including quasi-complete separation or collider stratification bias. Further studies are needed to explore these explanations and clarify the extent to which the observed association is causal.

Health systems should adopt a family-centered implementation strategy that promotes simultaneous vaccination of parents and children and reduces logistical barriers to receiving vaccines. Key components include physician recommendations to vaccinate the entire family, not just the individual seen during the visit, to support family-wide protection. Targeted messaging and workflows for same-visit vaccination should emphasize safety, convenience, and household-level benefits, and include reminders for parents to bring their children to scheduled appointments. Invite and recall systems should use proactive, culturally tailored outreach that prioritizes families with unvaccinated children or prior missed visits. Sibling bundling and household scheduling should allow all children in a family to be vaccinated during a single visit, supported by extended appointment times and flexible consent procedures.

These scalable interventions may leverage the strong link between parental and child vaccination identified in this study, turning parental uptake into a practical driver for improving pediatric influenza coverage. Public health campaigns and healthcare providers should emphasize the direct benefits of vaccinating children while recognizing the broader family context. Promoting parental and child vaccination together highlights the importance of protecting the entire household and can serve as a clear message to support vaccine uptake. In addition, addressing parental concerns and providing accurate, evidence-based information about vaccine safety and efficacy are central to effective vaccination strategies.

The coverage and likelihood of vaccination among children with chronic diseases were lower than expected, given that children with medical comorbidities are at higher risk for severe influenza infections and more adverse clinical outcomes (31). Parents of children with chronic illnesses may have heightened concerns about the efficacy and safety of vaccines (32). Indeed, vaccination might be perceived as a lower priority, mainly if parents are unaware of its importance. Healthcare providers, particularly those caring for children with comorbidities, play a vital role in promoting vaccination and reducing vaccine hesitancy. Their recommendations for influenza vaccination are vital for increasing vaccination rates (33). They should provide clear, evidence-based guidance on the importance of vaccination for children with chronic conditions, emphasizing the increased risk of severe outcomes following influenza infection, potential disease complications, and the vaccine’s effectiveness (34). Additionally, they should maintain open communication with parents, addressing specific concerns such as side effects or long-term safety.

Our findings suggest that low SES and being part of a minority group play a crucial role in influenza vaccine uptake among children. Prior research has consistently shown that children from lower-SES backgrounds are less likely to receive the influenza vaccine than their higher-SES counterparts (22, 35). Vaccine hesitancy in this group may stem from religious or cultural beliefs, limited exposure to public health messaging, and a general mistrust of external authorities. In addition, there may be a demand for accessible healthcare services tailored to their needs, such as reluctance to bring children to clinics outside their immediate neighborhood, a preference for walking-distance access, and limited availability during standard operating hours.

Arab and Ultra-Orthodox Jewish communities encounter overlapping structural and cultural barriers to pediatric influenza vaccination (36, 37). Many families live in peripheral or underserved areas with limited clinic hours and limited transportation, while larger households and caregiving demands make scheduling challenging (38, 39). Language and literacy gaps reduce the effectiveness of standard digital outreach, and lower trust in centralized authorities limits engagement with national health campaigns (40). Cultural norms and community structures shape when and how families seek care, often favoring local and trusted sources of information. These factors, compounded by socioeconomic disadvantage, weaken the impact of uniform vaccination strategies and call for tailored, community-based approaches.

These unique barriers highlight the need for culturally sensitive, community-based interventions to improve vaccination uptake in these populations. Strategies such as community-based outreach programs, school-located vaccination clinics, and culturally tailored education campaigns can help address these gaps and ensure more equitable access to vaccines (41). Feasible interventions within HMOs should focus on convenience, family-centered care, and cultural accessibility. Extending clinic hours and offering family appointments would allow parents and multiple children to be vaccinated together, supported by walk-in family clinics at community hubs or near religious centers. Mobile vaccination units can further increase reach. Multilingual messaging, community leader partnerships, and culturally tailored outreach through bilingual health workers can build trust and improve uptake (38). In-clinic prompts, bundled sibling appointments, and streamlined consent processes can ensure that every visit becomes an opportunity for vaccination.

This study has several limitations. As a retrospective cohort analysis, it is subject to biases related to data completeness and potential inaccuracies in electronic medical records. Selection bias is possible if children received influenza vaccines outside the MHS network and these vaccinations were not captured in the database. Parental smoking status may be misclassified or underreported, and clustering by family was not fully accounted for, which could underestimate within-family correlations in vaccination behavior. Despite adjustment for multiple sociodemographic and behavioral variables, residual confounding from unmeasured factors such as health literacy, healthcare access, or trust in medical authorities cannot be excluded. Data on parental attitudes and beliefs were unavailable, though these likely play an important role in vaccination decisions; a complementary qualitative study is planned to address this gap. The study population consisted exclusively of children aged 6 months to 6 years who were members of a large health maintenance organization in Israel. This may limit the generalizability of the findings to other populations, healthcare systems, or regions with different vaccination policies or demographic characteristics. Finally, as this is an observational study, causality cannot be inferred, and further prospective research is needed to validate these findings and explore additional factors influencing vaccination uptake.

## Conclusion

The strong association between parental and child vaccination status underscores the importance of a family-centered approach to vaccination promotion. Public health initiatives can more effectively increase vaccination coverage among young children by targeting parents and children in vaccination campaigns, ultimately contributing to better health outcomes and robust community immunity.

## Data Availability

The datasets presented in this article are not readily available because the data supporting this study are available from the corresponding author, but restrictions apply to the availability of such information. It was used under a license for the current study and is not publicly available. Data are, however, available from the authors upon reasonable request and with permission of the local ethics committee of MHS. Requests to access the datasets should be directed to shirleys2@tauex.tau.ac.il.
