# Supplementary material for: Family matters: influenza vaccination uptake and associated factors in children aged 6 months–6 years
Source: Front Public Health. 2025 Dec 19;13:1704497. doi: 10.3389/fpubh.2025.1704497 (PMC12757351; doi:10.3389/fpubh.2025.1704497)
Supplement: Supplementary file 1 [file Data_Sheet_1.pdf]

Supplementary Table S1-Comorbidities according to MHS registries

**Comorbidities**	No vaccine<br>N=251,193	Vaccine<br>N=41,885	p. value
Homebound	32 (0.0)	33 (0.1)	<0.001
Diabetes	91 (0.0)	11 (0.0)	0.311
Hypertension	55 (0.0)	11 (0.0)	0.501
Inflammatory bowel disease	13 (0.0)	6 (0.0)	0.044
**Any**	**187 (0.1)**	**61 (0.1)**	**<0.001**

Supplementary Table S2- Factors associated with influenza vaccine uptake, children aged 6 months- 6 years

	2022-2023	
	(95%CI) OR	p. value
Age	1.10 [1.09-1.11]	<0.001
Sex (female vs. male)	0.99 [0.96-1.02]	0.322
Socioeconomic status [1] (Med vs. Low)	1.31 [1.24-1.39]	<0.001
Socioeconomic status [1] (High vs. Low)	2.29 [2.16-2.44]	<0.001
Sector (Arab vs. Other)	0.70 [0.63-0.77]	<0.001
Sector (Ultra-Orthodox Jewish vs. Other)	0.71 [0.66-0.76]	<0.001
Parental smoking status (one parent vs. none)	0.85 [0.82-0.88]	<0.001
Parental smoking status (two parents vs. none)	0.80 [0.74-0.87]	<0.001
Comorbidity [2]	1.91 [1.25-2.91]	0.003
Parental vaccination [3]	41.90 [40.57-43.26]	<0.001
Number of doctor visits in the preceding year [4] (1-3 vs. none)	1.49 [1.35-1.65]	<0.001
Number of doctor visits in the preceding year [4] (4+ vs. none)	2.08 [1.89-2.28]	<0.001
Hospitalizations in the preceding year	1.15 [1.09-1.23]	<0.001

[1] Socioeconomic status defined by the Israel Central Bureau of Statistics [23]

[2] Comorbidities, as documented in the MHS registries, include homebound status, diabetes, hypertension, and inflammatory bowel disease

[3] Parental vaccination in the same influenza season, 09/2022- 03/2023

[4] Total number of pediatrician or primary care doctor visits registered in MSH in the year before the study, in person, by telephone, and/or digital encounters.

Supplementary Table S3- Factors associated with influenza vaccine uptake, children aged 18 months- 6 years

	2022-2023	
	(95%CI) OR	p. value
Age	1.20 [1.19-1.22]	<0.001
Sex (female vs. male)	0.98 [0.94-1.02]	0.249
Socioeconomic status [1] (Med vs. Low)	1.17 [1.09-1.25]	<0.001
Socioeconomic status [1] (High vs. Low)	1.64 [1.53-1.76]	<0.001
Sector (Arab vs. Other)	0.90 [0.81-1.00]	0.067
Sector (Ultra-Orthodox Jewish vs. Other)	0.82 [0.76-0.88]	<0.001
Parental smoking status (one parent vs. none)	0.91 [0.88-0.95]	<0.001
Parental smoking status (two parents vs. none)	0.94 [0.85-1.03]	0.157
Comorbidity [2]	1.38 [0.84-2.27]	0.210
Previous year vaccination	10.35 [9.98-10.74]	<0.001
Parental vaccination [3]	27.07 [26.03-28.14]	<0.001
Number of doctor visits in the preceding year [4] (1-3 vs. none)	1.26 [1.13-1.40]	<0.001
Number of doctor visits in the preceding year [4] (4+ vs. none)	1.43 [1.29-1.57]	<0.001
Hospitalizations in the preceding year	1.15 [1.06-1.24]	<0.001

[1] Socioeconomic status defined by the Israel Central Bureau of Statistics [23]

[2] Comorbidities, as documented in the MHS registries, include homebound status, diabetes, hypertension, and inflammatory bowel disease

[3] Parental vaccination in the same influenza season, 09/2022- 03/2023

[4] Total number of pediatrician or primary care doctor visits registered in MSH in the year before the study, in person, by telephone, and/or digital encounters.
